# Modeling approaches for early warning and monitoring of pandemic situations as well as decision support

**DOI:** 10.3389/fpubh.2022.994949

**Published:** 2022-11-14

**Authors:** Jonas Botz, Danqi Wang, Nicolas Lambert, Nicolas Wagner, Marie Génin, Edward Thommes, Sumit Madan, Laurent Coudeville, Holger Fröhlich

**Affiliations:** ^1^Department of Bioinformatics, Fraunhofer Institute for Algorithms and Scientific Computing (SCAI), Sankt Augustin, Germany; ^2^Bonn-Aachen International Center for Information Technology (B-IT), University of Bonn, Bonn, Germany; ^3^Quinten Health, Paris, France; ^4^Sanofi, Paris, France; ^5^Department of Computer Science, University of Bonn, Bonn, Germany

**Keywords:** pandemic, machine learning, artificial intelligence, agent-based-modeling, compartmental models

## Abstract

The COVID-19 pandemic has highlighted the lack of preparedness of many healthcare systems against pandemic situations. In response, many population-level computational modeling approaches have been proposed for predicting outbreaks, spatiotemporally forecasting disease spread, and assessing as well as predicting the effectiveness of (non-) pharmaceutical interventions. However, in several countries, these modeling efforts have only limited impact on governmental decision-making so far. In light of this situation, the review aims to provide a critical review of existing modeling approaches and to discuss the potential for future developments.

## Introduction

In December 2019, a new virus (SARS-CoV-2), causing a respiratory disease - later named COVID-19^1^, was discovered. At the time of the outbreak, many healthcare systems around the world were not well prepared for the pandemic that later emerged. While the virus was initially detected in China, measures to prevent its spread to other regions of the world were often hesitant and taken too late. Whereas compartmental spatio-temporal models of disease spread in epidemiology have been known in principle for a long time (1), many countries initially lacked robust and systematically collected surveillance data to which these models could be fitted. In general, it has been difficult to translate insights from modeling into actionable decision support for the government.

**Figure 1:**
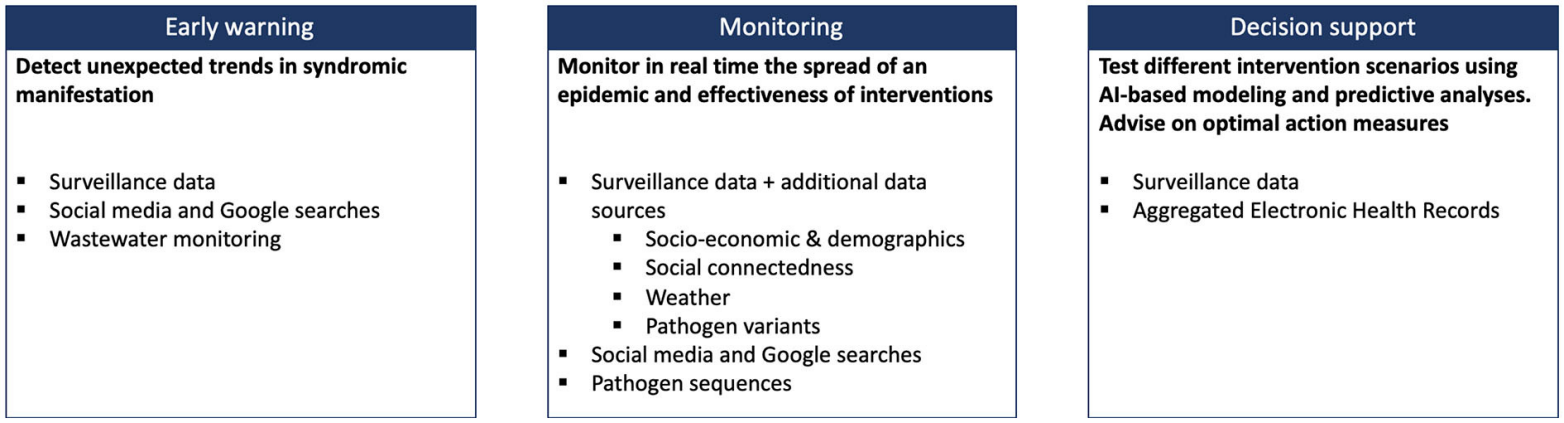
Overview of potential impact areas of population-level computational modeling for increased preparedness against pandemic situations, including relevant data sources.

Based on these considerations, the French-German collaborative project AIOLOS (Artificial Intelligence Tools for Outbreak Detection and Response) has recently started with the aim to strengthen the resilience of national healthcare systems against future outbreaks of respiratory infections^2^. More specifically, AIOLOS identifies three areas, where population-level computational modeling, including techniques from Artificial Intelligence (AI) and machine learning (ML), could potentially impact the preparedness against future pandemics based on various data sources (Figure 1):

1. early warning of a new outbreak,
2. monitoring the spatio-temporal spread of a disease,
3. predicting the impact and effectiveness of different interventions to support decision-making at scientific and policy levels.

This paper aims to review existing population-level computational modeling work in each of these areas. Our ambition is thus significantly different from published reviews, which solely focused on mathematical models of COVID-19 disease spread (2) or AI/ML algorithms for patient-level disease diagnosis and prognosis (3).

## Early warning

### Surveillance data

Health surveillance data is the traditional source of information for detecting a pandemic outbreak. The goal of respective computational approaches is to detect anomalies in a data stream consisting of discrete events, i.e., cases reported by doctors. For this purpose, several statistical tests have been suggested in the literature, including methods proposed by the Robert Koch Institute in Germany (4) and the Center for Diseases Control and Prevention in the USA (5), the Farrington method and its variants (6, 7) and Bayesian methods (4). Altogether, the R-package “surveillance” lists almost 20 algorithms for the early detection of pandemic outbreaks using surveillance data (8), covering three different scenarios:

1. spatio-temporal data of individual infectious events,
2. temporal event history of a defined set of individual units (e.g., specified households),
3. events aggregated over regions and time periods.

Due to data privacy concerns, typically only data of the last category are made publicly available and considered for governmental decision-making. A comparative simulation study pointed out elevated false positive rates for many algorithms with sensitivities ranging between 20 and 67% (9). Furthermore, a principal challenge is that traditional surveillance data in many countries are not systematically recorded in a fully automated and digitalized manner. Moreover, surveillance data in several countries do not cover several relevant aspects, such as hospitalization and ICU admission rates. Hence, this data could come too late for an early warning system. In response to this situation, several authors have thus proposed to systematically monitor wastewater for virus particles rather than waiting for reports by doctors (10, 11), and according measures are currently being implemented in the USA, Europe, and Israel. Noteworthy, Israeli researchers already used such an approach a few years ago to detect a silent polio outbreak (12, 13).

### Social media

Given the shortcomings of traditional surveillance data, several authors have more recently explored the potential of social media. Jain and Kumar (14) proposed a keyword extraction approach, in which they first used the term frequency-inverse document frequency (TF-IDF) technique, identifying relevant keywords from tweets, and secondly, used a linear discriminant analysis (LDA)-based classifier to find relevant keywords in newspaper really simple syndication (RSS) feeds. Subsequently, the relevant keywords were used to analyze tweets from the respective period, and machine learning classifiers were developed to filter out irrelevant tweets. They found that Support Vector Machines (SVMs) and a Naive Bayes classifier most accurately classified tweets (*F*_1_ = 0.77).

Lopreite et al. (15) performed statistical tests (Kolmogorov-Smirnov and Anderson-Darling) to compare the cumulative frequencies of pneumonia-related tweets from the winter seasons of 2018/2019 and 2019/2020 in selected European countries. They found an exceeding number of pneumonia-related postings in the winter season of 2019/2020 before the outbreak of COVID-19. In a similar direction, Mavragani (16) retrieved Google Trends data for the topic of “Coronavirus” and calculated Pearson correlation coefficients between Google Trends data and the respective categories of cumulative/daily cases/deaths. The results showed strong correlations of Google Trends data with COVID-19 cases and deaths in the examined European countries. The authors conclude that *information epidemiology* is a viable instrument to monitor the disease spread and identify regions in which cases have not yet peaked, hence contributing to an early warning system.

Going methodologically one step further, Yousefinaghani et al. (17) used a real-time anomaly detection approach utilizing the Seasonal-Hybrid Extreme Studentized Deviate algorithm (18) to identify the onset and peak of COVID-19 waves in Google Trends and Twitter data from the US and Canada. This study also evaluated the correlation between tweets and Google trends data with official COVID-19 case numbers. Pearson correlation analysis demonstrated a strong correlation between officially reported infected cases and the relevant posts and searches. Unlike other studies, the authors quantitatively prioritized COVID-19 symptoms in detecting disease trends. For example, “cough” and “fever” were better trend indicators compared to “tiredness” and “loss of smell.”

Broniatowski et al. (19) identified health-related, influenza-related, and case-reporting tweets with logistic regression, which were used with Google Flu Trends to predict influenza outbreaks at municipal and regional levels.

Further, Kogan et al. (20) used a Bayesian probabilistic model to develop an early warning algorithm for COVID-19 based on social media (Google Trends, Twitter, UpToDate), fever incidence rates, and predictions made by the global epidemic and mobility model (21), resulting in a time-to-event prediction. The algorithm was validated on COVID-19 surveillance data as well as incidence rates of influenza-like illness, demonstrating that an uptrend in COVID-19 infections could be predicted up to 7 days in advance with an accuracy of ~75%. Table 1 summarizes the techniques employed by the discussed papers.

**Table 1:** Included early warning studies.

**Studies**	**Data source**	**Technique(s)**
Höhle (4), Stroup et al. (5), Farrington et al. (6), Noufaily et al. (7), Meyer et al. (8), Lastra et al. (10), Maida et al. (11), Sharara et al. (12), Brouwer et al. (13)	Surveillance Data (health data, wastewater)	R package “surveillance,” anomaly detection, statistical tests
Jain and Kumar (14), Mavragani (16), Yousefinaghani et al. (17), Hochenbaum et al. (18), Broniatowski et al. (19), Kogan et al. (20)	Social Media (Twitter, Google trends, Newspaper feeds, UpToDate)	Keyword extraction, TF-IDF, anomaly detection, classifier (SVM, Naive Bayes), statistical tests (Kolmogorov-Sminrvov, Anderson-Darling, Pearson correlations), BM

TF-IDF, term frequency–inverse document frequency; SVM, Support Vector Machine; BM, Bayesian Model.

## Disease monitoring

### Spatio-temporal modeling of disease spread

There are different approaches for modeling the spatio-temporal spread of an epidemic situation described in the literature (see Tables 2–5):

- mechanistic compartmental models formulated as differential equation systems, which have been classically used in epidemiology (22, 26–33, 35–38, 40, 64),
- machine learning approaches, including Bayesian learning techniques (41–49),
- agent-based modeling approaches (50–55),
- hybrid modeling approaches combining several of the aforementioned techniques (39, 56, 58–63, 65).

**Table 2:** Included studies covering spatio-temporal monitoring of disease spread with compartmental models and their key aspects.

**Study**	**Key aspects**
Zhang (22)	Include factor for incubation time, immunity, and control efforts
Shaman et al. (23)	Use an EAKF to adjust (un)observable state variables
Leonenko and Ivanov (24)	Model dynamics of influenza outbreaks on city level
Osthus et al. (25)	Relate SEIR to state-space model and expand parameter vector
Aravindakshan et al. (26)	Estimate connection between NPIs and social mobility: used in the model
Bahri (27)	Splits between young and older population and estimates efficacy of NPIs
Bertozzi et al. (28)	Compare three basic models for different stages of pandemic
Chang et al. (29)	Introduce mobility networks between CBGs and POIs
Coudeville et al. (30)	Estimate effect of NPIs on industry decisions
Giordano et al. (31)	Model distinguishes between detected and undetected and among SOI
Götz and Heidrich (32)	Use registered deaths as parameter including a delay-term
Khan et al. (33)	Include detected and undetected cases and measure the effect of NPIs
Pei et al. (34)	Investigate spatial dynamic coupling across locations for asynchronous NPIs
Prague et al. (35)	Augment data to account for random effects and to increase accuracy
Coudeville et al. (36)	Study vaccination with different immunization programs
Humphrey et al. (37)	Introduce isolation compartment to study social distancing
Kheder et al. (38)	Introduce multiple discrete stages to account for multiple waves
Sartorius et al. (39)	Study different spatial patterns (e.g., of mortality) in small areas
Schüler et al. (40)	Implement effect of NPIs by using a piecewise constant transmission rate

EAKF, ensemble adjustment Kalman filter; SEIR, susceptible-exposed-infected-recovered; NPI, non-pharmaceutical intervention; CBGs, census block groups; POIs, points of interest; SOI, severity of illness.

**Table 3:** Included studies covering spatio-temporal monitoring of disease spread with machine learning and Bayesian models and their key aspects.

**Study**	**Key aspects**
Stojanović et al. (41)	Introduced a spatio-temporal kernel function
Al-qaness et al. (42)	Forecast for the upcoming days with a fair amount of data
Fong et al. (43)	Develop forecasting model with insufficient amount of available data
Mehta et al. (44)	Estimate outbreak probability on county level
Pavlyshenko et al. (45)	Investigated impact on stock market
Suzuki et al. (46)	Use binary classification to see if number of cases will exceed a threshold
Ibrahim et al. (47)	Implement urban characteristics and index for NPIs
Nader et al. (48)	Estimate growth rate depending on specific NPI
Yeung et al. (49)	Compared non-time series ML algorithms to model pandemic

NPI, Non-Pharmaceutical Intervention; ML, machine learning.

**Table 4:** Included studies covering spatio-temporal monitoring of disease spread with agent-based modeling approaches and their key aspects.

**Study**	**Key aspects**
Hoertel et al. (50)	Estimate impact of post-lockdown measures and introduce shielding of PAR
Hinch et al. (51)	Estimate effect of contact tracing with mobile app
Keer et al. (52)	Model by calculating probability of agent to change state at a timepoint
Staffini et al. (53)	Retrospectively study effect NPIs had and additional NPIs could have had
Colosi et al. (54)	Estimate reproduction numbers for different VOC in schools
Shattock et al. (55)	Analyze different NPI and vaccination strategies

PAR, persons at risk; NPI, non-pharmaceutical intervention; VOC, variance of concern.

**Table 5:** Included studies covering spatio-temporal monitoring of disease spread with hybrid models and their key aspects.

**Study**	**Key aspects**
Dandekar and Barbastathis (56)	Analyze NPIs in different countries to find effective reproduction number
Menda et al. (57)	Estimate dynamic transmission number with NN, allowing for multi-peaks
Silva et al. (58)	Build society with ABM and simulate different NPI scenarios
Capobianco et al. (59)	Combine ABM and SEIR with Markov model and RL for NPI planning
Wang et al. (60)	Combine spatial and temporal models
Watson et al. (61)	Predict deaths by relation between cases and population characteristics
Fritz et al. (62)	Use a GNN to include local mobility and connectedness data from Meta
Hadley et al. (63)	Modify transmission and hospitalization rates fitted to agent's characteristics

NPI, non-pharmaceutical intervention; NN, neural network; ABM, agent-based modeling; SEIR, susceptible-exposed-infected-recovered; RL, reinforcement learning; GNN, graph neural network.

#### Compartmental models

##### General principle

To model and understand the evolution of an epidemic, compartmental models are often used. The underlying idea is to distribute the population into several interconnected compartments. The relationship between these compartments is given by a system of differential equations. With given or estimated initial conditions this mathematical system can be solved at any point in time The foundation of today's compartmental models was formulated nearly a century ago (1). In their study, Kermack and McKendrick examined the evolution of various pandemics and established the commonly used susceptible-infected-removed (SIR) model which is based on three compartments:

- *S*(*t*) - The **susceptible** population, i.e., the part of the population that can become infected,
- *I*(*t*) - The **infected** population, i.e., the part of the population that has the disease and can transmit the disease to the susceptibles,
- *R*(*t*) - The **removed** or **recovered** population, i.e., the part of the population that has recovered from the disease and that is considered immune. (With *N* = *S*(*t*) + *I*(*t*) + *R*(*t*) being the total population.)

The dynamics of the SIR model get described by a set of ordinary differential equations (ODEs), which include two free parameters, β - the transmission rate and γ - the recovery rate:


$$\begin{array}{l} \frac{dS}{dt}=-\beta SI\\ \frac{dI}{dt}=\beta SI\;-\;\gamma I\\ \frac{dR}{dt}=\gamma I. \end{array}$$

Due to its simple nature, there are also some limitations and assumptions with this model. Here we will mention some of them. First, the population size is assumed to be constant, the birth nor the death rates are incorporated, and the model does not allow for people to become reinfected. Second, both the transmission and the recovery rates are constant. Third, the model assumes that the infected person becomes infectious immediately after getting infected, whereas in reality there is a latency period. Another assumption is that there is homogeneous mixing of the population, and no social networks and mobility are considered.

To account for some of its limitations, the archetypical SIR model can be extended to include an age structure or additional compartments, e.g., compartment E for the - by the virus-exposed - population (susceptible-exposed-infected-removed: SEIR), compartment D for the disease-deceased population or compartment H for the hospitalized population.

##### Applications to epidemic disease monitoring

There is a vast literature on compartmental disease models over the last 50 years (66). Examples include the successful modeling of several epidemic outbreaks, such as SARS (22) and influenza (23–25). However, the highly dynamic development of the COVID-19 pandemic with corresponding public intervention measures required extensions and modifications (26, 27, 30, 32, 36, 37, 40). For example, Götz and Heidrich (32) used the number of registered deaths by COVID-19 rather than the registered cases, with the idea to evade the dark figure of undetected cases, including a delay term to account for the time between infection and death. Bahri (27) split between a young population (age < 60 years) and an older population (age ≥60 years) stating that the younger population has more infections, while the older population is at higher risk, with a much higher death rate. Similarly, Coudeville et al. (30) introduced an age-stratified SEIR model to estimate how different scenarios affect industry decisions on different time scales. In another study, the authors further used this model to derive the potential effects of various immunization programs based on vaccination (36).

Aravindakshan et al. (26) used a compartmental model including social distancing and mobility as parameters. The authors further estimated the impact of different non-pharmaceutical interventions (NPIs) on social distancing including other covariates (e.g., weather, day of the week) in a linear regression model and used its coefficients for simulating different scenarios. Schüler et al. (40) included NPIs by using a piecewise constant transmission rate depending on the corresponding NPI and analyzed effects on the district level. Similarly, Humphrey et al. (37) estimated the effect of testing and tracing in combination with social distancing measures by introducing an isolation compartment, resulting in a modified transmission rate. Prague et al. (35) estimated several parameters of an extended SEIR model from data about the incident and hospitalized cases in France at a regional level *via* a non-linear mixed effects model while considering NPIs. Moreover, the model by Prague et al. considers the fact that only a fraction of the actually infected patients is counted in surveillance data.

Bertozzi et al. (28) studied the disease spread in several European countries, first looking at the exponential growth and the self-exciting branching process and then using a compartmental model, focusing on the impacts of social distancing, enabling them to model and understand different stages of the pandemic.

Khan et al. (33) modeled an NPI-dependent transmission rate. Chang et al. (29) modeled the disease spread in the ten largest US metropolitan areas using bipartite networks with time-varying edges for mapping the hourly movement of census block groups (CBGs) to specific points of interest (POIs). Then, each mobility network gets paired with an extended SEIR model with a corresponding transmission rate. To illustrate the spatial dynamic coupling across locations, Pei et al. (34) used a metapopulation SEIR model including daily work commuting and random movement among 3,142 US counties. Using inference, they studied the effect of asynchronous interventions across these locations in the US and performed counterfactual simulations to estimate the evolution of the disease spread by implementing NPIs at different times. To account for the fact that COVID-19 is a pandemic with several waves, Khedher et al. (38) introduced multiple discrete states into their model.

Sartorius et al. (39) developed a discrete-time SEIR model, which incorporated information about population density and mobility using a hierarchical Bayesian model. They estimated their model *via* full Bayesian inference (Markov Chain Monte Carlo sampling).

#### Machine learning models

In addition to compartmental models, machine learning techniques, including neural networks, have become popular approaches for modeling and predicting disease spread. Examples include models for the disease spread in China (43) and worldwide (47). Fong et al. (43) tried to overcome the problem of a small dataset by using a *polynomial* neural network with corrective feedback, while Ibrahim et al. incorporated urban characteristics and NPIs *via* a variational Long Short-Term Memory (LSTM) encoder.

In addition to neural networks, other machine learning techniques have been proposed as well: for example, Al-qaness et al. (42) combined an Adaptive Neuro-Fuzzy Inference System (ANFIS) with a flower pollination algorithm (FPA) using the salp swarm algorithm (SSA), creating the FPASSA-ANFIS model. Nader et al. (48) developed a Random Forest algorithm; other studies employed extreme stochastic gradient boosting (XGBoost) (44, 46). Yeung et al. (49) compared different classical machine learning regression methods (ridge, decision tree, Random Forests, AdaBoost, and Support Vector Machines) and found Random Forests and AdaBoost to perform best. In general, classical, non-time series machine learning models could predict future pandemic development rather accurately.

Pavlyshenko (45) used a Bayesian machine learning approach for modeling the global spread of COVID-19 and its effect on the stock market, while (41) additionally included the spatial aspect *via* a spatio-temporal kernel function.

#### Agent based models

Agent-based modeling (ABM) is a sub-field of Artificial Intelligence (AI). The idea in ABM is to simulate a set of software agents, which can interact with each other according to a defined set of rules. ABM approaches can implement many characteristics such as social contacts of individuals or sub-populations, disease characteristics (e.g., virus transmission rates, virus variants), patient characteristics (e.g., age, sex, comorbidities, and risk factors), mobility and contact networks (e.g., household, workplace, school, community, tourism), healthcare services (e.g., hospitalization, bed occupancy) and governmental regulations or NPIs.

In the literature, ABM approaches have been used on different scales. Staffini et al. (53) used socio-economic and disease-related information to study the spread of the SARS-CoV-2 virus and the influence of NPIs in Italy, Germany, Sweden, and Brazil. Shattock et al. (55) included risk groups and seasonal patterns in the transmission model and estimated the effect of various NPIs as well as vaccination campaigns on the pandemic evolution, hospitalization, and deaths in Switzerland. Colosi et al. (54) used an ABM approach to estimate school-specific reproduction numbers depending on the COVID-19 variants.

Various authors further extended these models by including demographic features as well as more profound contact networks - through deeper population mobility simulations - to simulate synthetic populations, the disease spread in this population, and the effect of a large set of NPIs (50–52). Hoertel et al. (50) focused on possible post-lockdown measures to reduce epidemic rebounds and therewith estimated the effect of protecting/shielding persons at risk; while Hinch et al. (51) and Kerr et al. (52) both developed a simulation platform, *OpenABM*, and *Covasim*, which enables to simulate the disease spread depending on various settings, including different NPIs.

#### Hybrid models

One of the main limitations of machine learning is the assumption of test data being drawn from the same statistical distribution as training data. This results in a major challenge if there is a covariate shift of test data relative to the original training data, e.g., due to NPIs, seasonal effects, new virus variants, or further unknown factors. Hence, the utility of conventional machine learning models in a highly dynamic situation such as the COVID-19 pandemic must be questioned. In this regard hybrid modeling approaches combining compartmental models and machine learning, or compartmental models and ABM approaches could provide an interesting alternative.

Several authors have explored hybrid models of the spatio-temporal disease spread in this regard: For example, Dandekar and Barbastathis (56) used a neural network to model the influence of NPIs on the compartment of infected patients. For model training, they employed the universal ODE approach, which combines neural networks with ODEs in a joint framework (67). Menda et al. (65) introduced a neural network to relax the assumption of a constant transmission rate. Their model is formulated as a non-Gaussian state-space system, which is estimated *via* Certainty-Equivalent Expectation-Maximization (57).

Wang et al. (60) combined their extended SIR model with spatial cellular automata (CA) and then introduced a Convolution Neural Network (CNN) paired with an LSTM recurrent neural network to learn the dynamical parameters of a compartmental model, which also includes the population of undetected or asymptomatic individuals.

Watson et al. (61) first used a probabilistic graphical model estimated *via* Bayesian inference to predict the velocity of cumulative cases. Moreover, they developed a Random Forest model to give daily projections and interval estimates for cases and deaths in different US states. Both models were then combined into a compartmental model to make forecasts of incidence rates.

A different type of hybrid model is presented by Fritz et al. (62). They combined a statistical spatial regression with a Graph Neural Network (GNN) incorporating social connectedness and co-location maps.

Several authors combined ABM approaches with SEIR models (58, 59, 63). Hadley et al. (63) derived the transmission and hospitalization rates depending on the agent's age, comorbidity status, and testing status to forecast the ICU bed demand. Silva et al. simulated a society (i.e., persons, houses, businesses, government, and healthcare systems) including a large set of social and demographic parameters and estimated different scenarios based on social distancing measures. Capobianco et al. introduced the *PandemicSimulator* including – besides a SEIR model – a moving and interacting society, a government that makes policy decisions, and optional testing and contract tracing strategies. They also suggested adding a hidden Markov model to adapt infection rates over time and a reinforcement learning (RL) layer to find the optimal policy to minimize the public health impact.

### Social media and internet searches

The epidemic spread has been shown to be correlated with search engine usage on the web in the past (68). Nowadays people also share their opinion on social media networking sites such as Twitter, Reddit, and Facebook. These opinions can also be utilized to track epidemic disease spread. Masri et al. (69) studied using tweets' time and geolocation data to improve the monitoring of the Zika virus (ZIKV) epidemic. The collected tweets were counted and compared with weekly data of the U.S. ZIKV cases, revealing a high Pearson correlation coefficient value of 0.67 by applying a 1-week lag on tweets. Adding this 1-week-lag tweet data to the case counts in an auto-regression prediction model improved the coefficient of determination (*R*^2^) from 0.61 to 0.74, which showed that tweet metadata is a significant predictor of future ZIKV cases.

Various authors have also used social media to support the surveillance and monitoring of an epidemic (70–72). Missier et al. (70) identified tweets related to dengue epidemics by classifying them into mosquito, sickness, and news-related classes. Chen et al. (73) created an ongoing collection of so far 123 million COVID-19-related tweets identified using various keywords and shared it with the research community for further analysis.

To better understand and model the trajectory of COVID-19 in the US, Klein et al. (74) manually annotated 10,000 pre-filtered tweets into three COVID-19 associated classes (probable, possible, and other cases) and used Bidirectional Encoder Representations from Transformers (BERT) to automatically classify tweets. The classifier achieved an F_1_ score of 0.64 for differentiating three classes. Given that “probable” or “possible” tweets were primarily distributed in the states reporting COVID-19 cases and posted before the first confirmed case, the model could successfully identify candidate COVID-19 cases and high-risk regions.

Similarly, Liu et al. (75) collected COVID-19-related Reddit posts from North Carolina, which showed a similar trend of observed confirmed cases and deaths as to the government data. They further classified these posts while performing NER to obtain mitigation types (such as distancing, disinfection, personal protective equipment) and detection types (such as symptoms, testing) and analyzed for a certain time period the change of people's sentiments toward masks in these posts. For disease monitoring, Magge et al. (76) built a system to collect symptoms and disease mentions from social media platforms and normalized them to unified medical language system (UMLS) terminology. Using deep learning methods (such as BERT and RoBERTa) that were trained on multiple available corpora (such as TwiMed, MedNorm, DS-NER), they achieved an F_1_-score of 0.86 and 0.75 on DailyStrength and Twitter datasets, respectively. They also applied their system on Twitter posts to collect COVID-19 symptoms.

Users also share their opinions on COVID-19 measures on Twitter by supporting, refuting, or just commenting on them (77). These opinions from German-speaking countries were manually labeled, and Beck et al. utilized predictions by transformer-based models. Jalil et al. (71) performed sentiment analysis on tweets' text to classify them into positive, negative, and neutral. For the analysis, they used the *COVIDSenti* dataset (78) and reached the highest accuracy of 96.66% with the proposed Multi-depth DistilBERT method. Table 6 provides an overview of the use of social media and internet searches for disease monitoring.

**Table 6:** Included studies focusing on disease monitoring *via* mining of social media and internet searches.

**Study**	**Aim**	**Technique(s)**
Ginsberg et al. (68)	Analyzed search queries to monitor influenza-like illness	Linear model
Missier et al. (70)	Compared methods for detecting disease related tweets	SC and LDA
Jahanbin et al. (72)	Developed text-mining method for disease related tweets	FAEMC-ID
Masri et al. (69)	Used tweets to predict future ZIKV cases	Auto-reg. prediction
Chen et al. (73)	Collected COVID-19 related tweets	Keyword collection
Klein et al. (74)	Modeled the COVID-19 disease spread with associated tweets	BERT
Beck et al. (77)	Analyzed tweets about reaction to COVID-19 measures	Ger-BERT
Liu et al. (75)	Analyzed tweets for cases and deaths and people's sentiments	NER
Magge et al. (76)	Monitored COVID-19 disease spread and collected symptoms	BERT and RoBERTa
Jalil et al. (71)	Analyzed tweets for people's sentiments and classified them	DistilBERT

ZIKV, Zika-virus; SC, supervised classification; LDA, linear discriminant analysis; FAEMC-ID, fuzzy algorithm for extraction; monitoring; and classification of infectious diseases; Auto-reg., auto-regression; BERT, bidirectional encoder representations from transformers; Ger-BERT, German bidirectional encoder representations from transformers; NER, named entity recognition; RoBERTa, robustly optimized bidirectional encoder representations from transformers approach; DistilBERT, distilled bidirectional encoder representations from transformers.

### Pathogen sequences

Pathogens are, like any organism, under evolutionary pressure and will thus mutate to optimize their adaptation to the human host. Accordingly, different pathogenic variants will occur over time. Deep learning approaches have recently been introduced to identify such variants during sequencing (79). In addition, phylogenetic tree inference, a classical approach from computational biology based on a sequence alignment followed by a statistical tree inference (either maximum likelihood or Markov Chain Monte Carlo) with a dedicated likelihood function (80), is often used. Incorporation of spatio-temporal information into the construction of phylogenies could potentially provide important information on the spread of virus variants. Still, phylogenies are not only informed by pathogen sequences, but also by external factors, such as the sampling process, the proportion of the pathogen genome sequenced in each sample, the quality of the sequence data, and the mutation rate of the pathogen itself (81).

Several authors have suggested approaches to construct temporal phylogenies (82–84) and applied this strategy to SARS-CoV-2 (85–87). More recently, Didelot et al. (88) showed that transmission events between hosts could be estimated by coloring different hosts in a phylogenetic tree reconstruction. Müller et al. (89) extended phylogenies to networks by incorporating recombination events and applied this strategy to influenza.

New variants may influence the transmission rate of a pathogen. Davies et al. (90) first retrospectively estimated the lineage-dependent growth rates of SARS-CoV-2. Based on that, they further calculated the expected competitive advantage of a new lineage and predicted the impact on the reproduction and the transmission rates *via* a discrete-time compartmental spatio-temporal disease model.

## Decision support

### Healthcare resource planning

Modeling can not only help to alert and monitor a pandemic situation, but forecasts generated by corresponding models can also give guidance on necessary actions. Therefore, there is no clear boundary between early warning, monitoring, and decision support.

One important aspect of decision support is the management and planning of available public healthcare resources. In this regard, Ivorra et al. (91) developed a compartmental model for China, in which they included the hospitalization rate. With the help of their model, they estimated and planned the demand for clinical beds. With a similar ambition in mind, Hadley et al. (63) proposed an agent-based modeling approach. Lorenzen et al. (92) developed a machine learning model (Random Forest) using electronic health records of more than 40,000 patients in Denmark, which predicted the number of ICU admissions and ventilator use. Kandula et al. (93) developed a compartmental model for predicting influenza hospitalization rates using Google search trends. Moa et al. (94) proposed a linear model to forecast the overall severity of an influenza season in Australia based on only five parameters.

### Planning and evaluating NPIs

In addition to healthcare resource planning a further aspect of modeling is to support the planning and evaluation of NPIs. In this context three different types of studies have been conducted (see Table 7):

- those that retrospectively evaluate the effects of NPIs (26, 27, 31–34, 40, 48, 49, 56, 60, 97, 98),
- those that make forecasts on the effects of a specified NPI in the sense of scenario planning (26, 31, 35, 38, 50–55, 58, 96),
- and those that develop methods for optimal control policy identification (59, 100–104).

**Table 7:** Included studies covering decision support.

**Studies**	**Technique(s)**
**Healthcare resource planning**	
Ivorra (91), Kandula et al. (93)	CM: including or predicting hospitalization rates
Moa et al. (94)	Linear model
Hadley et al. (63)	Agent-based modeling
Lorenzen et al. (92)	Random Forest using electronic health records
**NPI evaluation**	
Schüler et al. (40), Aravindakshan et al. (26), Khedher et al. (38), Giordano et al. (31), Prague et al. (35), Dandekar and Barbastathis (56)	CM: introducing NPI effect on transmission rate and reproduction number
Mader and Rüttenauer (95)	SCT: analyze effect of vaccinations
**NPI scenario planning and forecasts**	
Khedher et al. (38), Giordano et al. (31), Prague et al. (35), Kissler et al. (96)	CM
Staffini et al. (53), Shattock et al. (55), Colosi et al. (54), Hoertel et al. (50), Hinch et al. (51), Kerr et al. (52), Silva et al. (58)	ABM and hybrid ABM
Flaxman et al. (97)	Bayesian hierarchical model
Yeung et al. (49), Nader et al. (48), Barros et al. (98), Haug et al. (99)	ML
**NPI development**	
Kwak et al. (100), Colas et al. (101), Khadilkar et al. (102), Padmanabhan et al. (103), Chadi and Mousannif (104)	CM and RL: including health and economic costs
Capobianco et al. (59)	Hybrid ABM and RL

CM, compartmental models; NPI, non-pharmaceutical intervention; SCT, synthetic control technique; ABM, agent-based modeling; ML, machine learning; RL, reinforcement learning.

Retrospective evaluation of NPIs is generally challenged by the fact that NPIs are highly heterogeneous. Historically, often several NPIs have been applied at the same time, and there is neither a control group nor any kind of randomization. Systematic differences across countries in terms of demography, population density, climate, or cultural aspects complicates using of one country as a control for another one, even if typical statistical matching or weighting techniques known from observational studies are applied. Moreover, there is the question of the corresponding outcome to consider, given that observed incident cases will depend on the applied test strategy and thus underestimate the true number of infected people.

One type of approach has been to try to associate NPIs with the spatio-temporal modeling of disease spread, e.g., by introducing the NPI effect on the transmission rate and reproduction number in a compartmental model (26, 31, 35, 38, 40, 56). Correspondingly, authors have then used such models to make scenario forecasts, e.g., regarding the effect of social distancing (31, 35, 38, 96). Also, other types of spatio-temporal disease spreading models have been used for the same purpose, such as ABM approaches (50–55, 58), Bayesian hierarchical modeling (97), and machine learning (48, 49, 98, 99). The work of Yeung et al. specifically investigated the influence of socio-cultural aspects on the growth rate of COVID-19 incidences in 114 countries. The work by Barros et al. considered causal machine learning techniques.

Also, more traditional statistical analysis approaches have been applied recently, such as the synthetic control technique (95), which uses incident case numbers from the same country in the treatment and control group, depending on when an NPI has been put in place. Additionally, Mader and Rüttenauer analyzed the effect of vaccinations.

To find optimal control policies, offline RL strategies have been proposed by several authors. While Kwak et al. (100) solely relied on deep learning and only focused on health aspects, other studies (101–104) focused on a hybrid modeling strategy incorporating an extended SEIR compartmental model for predicting potential NPI effects. Moreover, the latter studies incorporated the economic costs of NPIs as well. Finally, Capobianco et al. (59) combined their hybrid ABM approach with offline RL to optimize the reopening policies.

## Discussion

Statistical tests have been used traditionally to detect outbreaks based on surveillance data. Recent years have witnessed an increasing use of other data sources, such as social media and internet searches. Even though such data types are likely to contain relevant signals, these are most likely biased toward certain user communities. Hence, early warning signals detected *via* “digital traces” should be seen as a complement to traditional surveillance data, but not as a replacement.

Regarding the monitoring of pandemics, specifically, the existing modeling efforts for COVID-19 have highlighted numerous challenges, such as the unknown number of truly infected persons (due to limitations of tests and test strategies, or due to asymptomatic disease) and the dependency on the spatio-temporal spread on external factors, such as NPIs and the compliance to those measures, weather, population density, and socio-economic aspects. Hence, many authors have extended traditional epidemiological compartment models and combined them with statistical inference and machine learning techniques, partially resulting in hybrid neural network /compartmental modeling approaches. While these are clear advancements, it should be seen that the spatio-temporal spread of an infectious disease is generally determined by a complex interplay between a pathogen (e.g., its genetic adaptability), individual (e.g., genetic variants, disease history, lifestyle, socio-economic conditions), society (e.g., testing strategy, vaccination rate, NPIs and compliance to those, population density) and environment (e.g., climate, weather). NLP techniques could help at this point to mine social media and news articles to complement surveillance data and to gain an understanding of the sentiment of the population with respect to specific NPIs, while at the same time taking into consideration the biases of this type of data and the principally limited accuracy of text analytics as such. Altogether, further developments of modeling approaches are needed, which better combine data modalities across all relevant scales, i.e., ranging from the pathogen up to the environment level. This, however, will in turn require better availability, integration, and accessibility of necessary data, including electronic health records. The investment into such a data infrastructure is thus a prerequisite to making significant progress on the modeling side.

Models will only have an impact if they can support the human decision process. In recognition of this fact, several authors have tried to support scenario planning by associating NPIs with the predicted spatio-temporal development of the disease, or by forecasting healthcare resources and economic impact. While forecasts under the scenario of no further taken action might be improved by considering the aspects mentioned above for spatio-temporal modeling, predicting the effect of an NPI is principally challenged by several aspects: (i) The NPI could be new and thus there is no direct historical comparison, and (ii) there is always a lack of a proper control group, i.e., it is not possible to perform a study akin to a Randomized Clinical Trial. RL techniques are thus generally challenged by this inability to experiment with a new policy. It is thus unlikely that decision-makers would immediately trust the recommendation of an optimal NPI estimated by an RL algorithm. A better approach might hence be to offer a ranking of the predicted effectiveness of multiple NPIs together with the estimated economic costs, which should not be neglected.

## Conclusion

In response to the ongoing COVID-19 pandemic, many countries currently review their strategies to be better prepared against future outbreaks. One important aspect in this context is to invest in data analytical capabilities, including modeling. Computational modeling approaches could help to earlier detect an outbreak, monitor the spatio-temporal spread, and to support the decision-making process by governmental authorities.

In this paper, we reviewed the diversity of existing modeling approaches for all three areas. Of course, each model is adjusted to a specific healthcare-related question by fitting it to particular data. In conclusion, models for early outbreak detection as well as spatio-temporal disease spread could be further improved by better combining and integrating data modalities across multiple scales. The ongoing COVID-19 pandemic in this context provides a “global laboratory” with the opportunity to retrospectively validate existing techniques as well as develop new ones. At the same, there is a need for funding bodies and governmental decision-makers to invest in corresponding data ecosystems. Models are likely to increase their impact on decision-making if they become more accurate and are at the same time explainable. Showing point estimates of a black-box model without highlighting epistemic uncertainties or providing further explanations of the most influential features is thus discouraged.

## Author contributions

Conceptualization, methodology, supervision, project administration, and funding acquisition: HF. Data curation, formal analysis, visualization, investigation, validation, and writing—original draft: JB, DW, NL, SM, and HF. Writing—review and editing: JB, DW, NL, MG, NW, ET, LC, SM, and HF. All authors contributed to the article and approved the submitted version.

## Funding

This work has been supported by the AIOLOS (Artificial Intelligence Tools for Outbreak Detection and Response) project. The project was supported by the French State and the German Federal Ministry for Economic Affairs and Climate Action (grant number 01MJ22005A) and the French Ministry of Economy and Finance. Ce projet a été financé par le gouvernement dans le cadre de France 2030 in the context of the Franco-German call on Artificial Intelligence technologies for risk prevention, crisis management, and resilience.

## Conflict of interest

Authors NL, NW, and MG are employees of the commercial company Quinten-Health. Authors ET and LC are employees of the commercial company Sanofi. None of the afore mentioned companies had any influence on the scientific content presented in this paper. The remaining authors declare that the research was conducted in the absence of any commercial or financial relationships that could be construed as a potential conflict of interest.

## Publisher's note

All claims expressed in this article are solely those of the authors and do not necessarily represent those of their affiliated organizations, or those of the publisher, the editors and the reviewers. Any product that may be evaluated in this article, or claim that may be made by its manufacturer, is not guaranteed or endorsed by the publisher.

## Footnotes

^1^ https://www.who.int/emergencies/disease-outbreak-news/item/2020-DON229

^2^ https://www.digitale-technologien.de/DT/Redaktion/EN/Standardartikel/Internationale_Koop_Projekte/Frankreich/ki_innovationsprojekte_de_fr_projekt_aiolos.html
